# The influence of motion quality on responses towards video playback stimuli

**DOI:** 10.1242/bio.011270

**Published:** 2015-05-11

**Authors:** Emma Ware, Daniel R. Saunders, Nikolaus F. Troje

**Affiliations:** 1 Department of Psychology, Queen's University, 99 University Ave, Kingston, ON K7L 3N6, Canada; 2 Centre for Neuroscience Studies, Queen's University, 99 University Ave, Kingston, ON K7L 3N6, Canada; 3 Wellesley Institute, 10 Alcorn Ave #300, Toronto, ON M4V 3B1, Canada; 4 Centre for Brain/Mind Sciences, University of Trento, via Calepina, 14-38122 Trento, Italy; 5 Department of Biology, Queen's University, 99 University Ave, Kingston, ON K7L 3N6, Canada; 6 Canadian Institute for Advanced Research, 180 Dundas St W, Toronto, ON M5G 1Z8, Canada

**Keywords:** Video playback, Social perception, Biological motion perception, Visual communication, Presentation rate

## Abstract

Visual motion, a critical cue in communication, can be manipulated and studied using video playback methods. A primary concern for the video playback researcher is the degree to which objects presented on video appear natural to the non-human subject. Here we argue that the quality of motion cues on video, as determined by the video's image presentation rate (IPR), are of particular importance in determining a subject's social response behaviour. We present an experiment testing the effect of variations in IPR on pigeon (*Columbia livia*) response behaviour towards video images of courting opposite sex partners. Male and female pigeons were presented with three video playback stimuli, each containing a different social partner. Each stimulus was then modified to appear at one of three IPRs: 15, 30 or 60 progressive (*p*) frames per second. The results showed that courtship behaviour became significantly longer in duration as IPR increased. This finding implies that the IPR significantly affects the perceived quality of motion cues impacting social behaviour. In males we found that the duration of courtship also depended on the social partner viewed and that this effect interacted with the effects of IPR on behaviour. Specifically, the effect of social partner reached statistical significance only when the stimuli were displayed at 60 *p*, demonstrating the potential for erroneous results when insufficient IPRs are used. In addition to demonstrating the importance of IPR in video playback experiments, these findings help to highlight and describe the role of visual motion processing in communication behaviour.

## INTRODUCTION

While morphological cues such as size, shape, colour, texture and brightness can all bear meaning in the social world, an animal's behaviour – the way that animal moves as it interacts with its environment – is commonly regarded as its most influential visual feature (Nakayama, 2010; Nicoletto and Kodric-Brown, 1999; Rosenthal et al., 1996; Shimizu, 1998). For birds especially, vision is highly tuned to conspecific movement and, in many cases, dynamic motion cues appear to be more behaviourally relevant than morphological cues alone (Bird and Emery, 2008; Byers et al., 2010; Shimizu, 1998). The pigeon (*Columbia livia*) is a case in point: pigeons interact with a rich repertoire of visual signals, each adapted to incite action in others (Dittrich et al., 1998; Frost et al., 1998; Jitsumori et al., 1999; Shimizu, 1998). It has previously been shown that male pigeons will respond with courtship behaviour towards video images of females much more vigorously when these images are presented in motion rather than still (Shimizu, 1998). Pigeons can discriminate between individual conspecifics when their images are presented in motion, but not when they are shown still (Jitsumori et al., 1999). Further, pigeons can discriminate conspecific actions (such as pecking and walking) from ‘point light displays’; stimuli that contain only motion cues and are stripped of all other visual qualities (Dittrich et al., 1998). For species like the pigeon that employ body movement in communication it can be assumed that the visual system is highly specialized to detect, identify and evaluate complex gestures, actions and even movement ‘styles’ from motion cues alone (Yamazaki et al., 2004).

The field of video playback research has developed video as a tool to study visual communication in non-human animals. By using video images in place of live-acting social partners, researchers can control and manipulate the visual presentation of social stimuli. By observing a subject's natural reaction towards these stimuli, one can begin to understand the complex meaning of visual signals. Evans and Marler (Evans and Marler, 1991) were among the first to employ these methods for studying social behaviour. They used video playbacks of female hens (*Gallus domesticus)* to show the influence of their presence on male alarm calling behaviour. Since then, video playback methods have been successfully employed for studying visual communications in a diverse variety of animal species, including invertebrates (Clark and Uetz, 1990), fish (Kodric-Brown and Nicoletto, 2001), reptiles (Ord and Evans, 2002) and birds (Shimizu, 1998; Galoch and Bischof, 2007).

A pervasive problem with video playback studies, however, is that their interpretation relies on the assumption that subjects perceive and respond to video the same as they would to a live social partner (D'Eath, 1998). Unfortunately, video has the potential to induce visual artifacts, especially for the non-human viewer, given that the technology is specifically engineered to satisfy the human eye. To uphold the successes and the progress of video playback research, it is essential to remain critical about these methods. Just because an animal reacts to a video image, and varies its behavioural response according to some experimental manipulation of the stimulus does not guarantee that the animal is reacting exactly as it would to those same variations in a live natural social stimulus. Any unnatural perceptual differences between the video image and the real thing could result in subtle changes in the subject's behavioural response variation towards social stimuli.

In this paper, we present, discuss and study one such video artifact that could impact the results of video playback experiments: the motion quality of the video stimulus. We use the pigeon and its courtship behaviour to study the impact of motion quality on behavioural responses towards moving images of opposite sex partners. To give context, we briefly review some better known video artifacts.

**List of abbreviations**

IPR: image presentation rate
*p*: progressive
*i*: interlaced
TCWD: total circle walking duration
CFF: critical flicker frequency
CSF: critical sampling frequency
LCD: liquid crystal display
PAL: European Phase Alternating Line television standard
NTSC: National Television System Committee television standard

Depth cues derived from binocular stereopsis and motion parallax are lacking in standard video images. Given the laterally placed eyes of the pigeon, and the small interocular distance between them, stereopsis probably plays only a minor role in pigeons anyway, specifically at distances beyond the reach of their beak. The more important depth cue is likely to be motion parallax, which is impossible to simulate given video data from a single stationary camera. While the misrepresentation of depth cues is essentially unsolvable, at least for natural video, most researchers believe that, if the proper precautions are made to minimize and control these artifacts, they should not severely affect the validity of most studies (Oliveira et al., 2000).

Colour cues filmed by a video camera and displayed on a screen likely do not appear natural to pigeons. Both the sensors used on video cameras and the screens on which recordings are displayed are designed such that the spectral content they produce stimulates the human visual system in the same way as the spectra that the depicted objects emit in the real world; these two spectral distributions are metameric. What is metameric to the human colour vision system is unlikely to be metameric to the pigeon colour vision system, which consists of four different photoreceptor types with absorption spectra that are profoundly different from the ones found in humans (Kelber et al., 2003). However, it seems that colours that must appear false to the pigeon visual system do not prevent birds from responding to video displays in natural ways (Shimizu, 1998; Cuthill et al., 2000).

Social interactivity is lacking in video stimuli. In real-world interactions a social partner will react with a characteristic pattern of social contingency, timing and spacing toward the actions of the subject animal (Watson, 1966). The interaction is a two-way dialogue, which is incomplete without the full reciprocal participation of the social partner.

There is also the problem of non-linear interactions between video artifacts and the behavioural context set up by the experimental variables [for review see (Fleishman and Endler, 2000)]. The impact of a video artefact may vary based on the experimental condition into which the subject is placed. What an animal attends and responds to when interacting with a conspecific depends on a number of social factors, such as the sex of the partner, the age, attractiveness, physical appearance or familiarity of that partner. For example, a female pigeon might attend to colour cues if the males she sees is a large mature male that she finds attractive, whereas she might not attend to those cues otherwise. In this case, a perceptual distortion of colour could alter the subject's responses for one male partner but not for another less attractive male, therefore producing a skewed pattern of experimental results across partners.

It is especially important to be vigilant for potential perceptual differences in something as fundamental as motion quality. Unnatural motion cues, being critical to social behaviour and communication, could fundamentally distort the meaning of a social signal. The mechanisms by which the complex motion patterns of animal signals are perceptually encoded and reacted to behaviourally are not well understood. It is important that the video motion patterns that we use to re-create and study these processes are accurate representations of the real live thing.

In this study, we use the pigeon and its courtship behaviour to explore the effect of video motion quality on the pigeon's responses to social cues presented on video. We manipulate a technical parameter called image presentation rate (IPR), the rate at which images are sampled by a camera and displayed on a screen. As will be described further below, changes in IPR have the effect of varying how smooth (high IPRs) or jerky (low IPRs) the motion appears; IPR can also have more complex perceptual effects impacting the ability to resolve details of rapid movements [e.g. see (Straw, 2003)]. We also investigate how the effect of IPR interacts with any effect that different social partners may have on subject behaviour.

### Image presentation rate and motion on video

A theoretical threshold mechanism can be applied to describe the relationship between IPR variation and the perception of motion, which is called the critical sampling frequency (CSF) (Watson et al., 1986). When the sampling and presentation rate of successive images exceeds CSF, the observer will see the illusion of smooth movement. If the video's IPR is below the subject's CSF, the sampled motion will appear distorted. The CSF threshold reflects the temporal resolving properties of an observer's motion perception mechanisms.

Previous research has shown that CSF is dynamic, variable and sensitive to variations in stimulus characteristics as well as the visual environment (McDonnell et al., 2007; Watson et al., 1986). In a series of psychophysical experiments using simple moving line stimuli, a state space may be described as ‘the window of visibility’ (Watson et al., 1986), within which sampled motion is perceived as smooth and continuous, rather than jerky. The boundaries of this theoretical window, representing CSF variation, are a function of the object's velocity and spatial frequency, as well as the observer's spatial and temporal acuity. While Watson and colleagues’ theoretical model helps frame the present work, it is impossible to apply a single ‘window of visibility’ concept directly to complex video stimuli. More directly related to the present work, McDonnell et al. (McDonnell et al., 2007) investigated variation in human CSF by varying the IPR (referred to as pose update rate in their paper) of computer generated animated human walking stimuli. Their aim was to understand how to make an informed choice about the balance between information investments in IPR and other image qualities to optimize the overall quality of the human observer's experience viewing animations. They manipulated the animated characters’ linear walking velocity, step cycle rate, group size, motion complexity (walking vs. running etc.) and clothing type. Then they asked observers to discriminate between smooth and jerky motion. The participants’ CSF varied from 12 to 27 poses per second and depended on the animated walkers’ gait cycle rate, linear velocity, action type, motion complexity and group size. Slower simpler movements and smaller group sizes generally required fewer image updates. These findings highlight the dynamic nature of CSF as depending on a number of complex image parameters that are likely to vary in videos of social stimuli.

To further identify the mechanisms underlying CSF threshold variation, the Nyquist sampling theorem is thought to describe low level filters for motion processing in the brain (Burr, 1981). The Nyquist theorem states that visual sampling must occur at twice the object's highest motion frequency to accurately represent its motion. For example, if the visual system (or a video camera) samples the environment at 30 Hz, the highest motion frequency that can be represented in the observer's perception is 15 Hz. Higher frequency motion components appear distorted in various ways, such as in temporal aliasing, where temporal signal components not present in the original display are introduced. Aliasing can result in the perception of multiple simultaneous images [called ghosting, (Straw, 2003)], or the wagon wheel effect, where apparent motion can reverse with respect to the actual movement direction.

It is important to clarify the distinction between motion distortions caused by low image sampling and motion distortion caused by screen flicker, as these are easily confused. Screen flicker occurs on older CRT screens. At each point in time, only one pixel on the screen is hit by the cathode ray that triggers the screen pigments to light up. At all other times this point remains dark. The refresh rate of the monitor is determined by the number of times per second that the cathode ray runs through the whole screen to light it up. As with motion perception, a theoretical threshold mechanism, known as the critical flicker frequency (CFF), can be applied to flicker perception, and this threshold can be used as a proxy to describe the temporal resolution of the visual system. If the monitor's refresh rate is lower than the observer's CFF, flickering light becomes perceptible to the observer. Depending on light intensity and the adaptation state of the observer, critical flicker frequency ranges between 3 and 63 Hz in humans (Simonson and Brozek, 1952). When the observer sees flicker, the discontinuity of motion that occurs is akin to observing dancers moving under a strobe light. The disturbance is not due to impoverished motion per se. Rather, the image is being masked by luminance variations which are visible not only in sequences that contain motion, but in still images, too. On the other hand, motion distortions arising from insufficient IPR are akin to what one might experience when viewing a low budget animation or Claymation, in which every single pose must be drawn (or moulded) separately and the IPR is usually low to save time and resources. The resulting motion appears distorted because of slow pose updates relative to the rate of movement, but there is no flickering light on the image.

CFF, rather than CSF, has been a primary focus of scientific inquiry. We know that many animals, particularly diurnally active, fast-flying birds, have relatively high CFF rates (e.g. Pigeons, 60 to 145 Hz; Powell, 1967; Hens, *Gallus gallus*, 105 Hz; Nuboer et al., 1992) and probably see flicker on older CRT systems which refresh at 50 Hz (European Phase Alternating Line television standard, PAL standard) or 60 Hz (National Television System Committee television standard, NTSC standard). The perception of screen flicker has often been suggested as a primary reason for failed attempts at eliciting natural response behaviours from animal subjects towards video (Ikebuchi and Okanoya, 1999; Railton et al., 2010; Schlupp, 2000). The deleterious effect of flicker on response behaviour is quite possibly due to the fact that flicker breaks up the visual motion illusion and therefore distorts a powerful cue for triggering behaviour (Schlupp, 2000). Fortunately, with the advent of liquid crystal display (LCD) monitors, a newer technology illuminated by a constant backlight, flicker is no longer a problem (Ikebuchi and Okanoya, 1999). LCD screens potentially introduce a new artifact, polarized light, but there is little evidence that birds are able to detect it (Muheim, 2011).

We know less about CSF but we can assume that species that have ‘faster’ vision than our own, such as diurnally active flying birds, should also be expected to possess higher CSF thresholds for smooth motion perception. Virtually no studies have directly measured CSF in a non-human species. Interestingly the only study we are aware of was conducted over 50 years ago, well before the first video playback research emerged. Hans Lissman, a distinguished German physiologist, placed Siamese fighting fish (*Betta splendens Regen*) inside a rotating wheel that contained evenly spaced slits revealing a cylindrical mirror that reflected the fish's own image (Lissmann, 1932). When the wheel was rotated very slowly, so that motion in the mirror could be observed while each slit passed, the fish responded aggressively to its own moving image. As the wheel rotated faster, the moving image became broken and the fish stopped responding, until it reached a speed of approximately 30 to 35 images per second. Then the fish started behaving aggressively again, towards its own image. The results imply that the perception of smooth movement in the mirror reflection was critical for triggering a natural behavioural response and that an IPR between 30 and 35 Hz represents the Siamese fighting fish's CSF under experimental conditions.

Could it be that video playback researchers have been presenting undersampled motion stimuli to their animal subjects? The standard IPRs used until recently include 24 *p* (film), 25 *p,* 30 *p*, 50 *i* (European Phase Alternating Line television standard, PAL) and 60 *i* (National Television System Committee television standard, NTSC), where *p* indicates progressive format and *i* indicates interlaced format. In progressive video, the IPR indicates how many full image frames are displayed each second. For example, video displayed at 30 *p* shows 30 full image frames per second. In interlaced video, two image fields are displayed within each video frame, where each field contains every second line of a new image. Thus, video displayed at 60 *i* shows 30 frames, containing 60 separate image fields per second. Since each field of interlaced video is shot at two different time points, temporal resolution and motion perception become enhanced without consuming extra bandwidth.

**Fig. 1.**
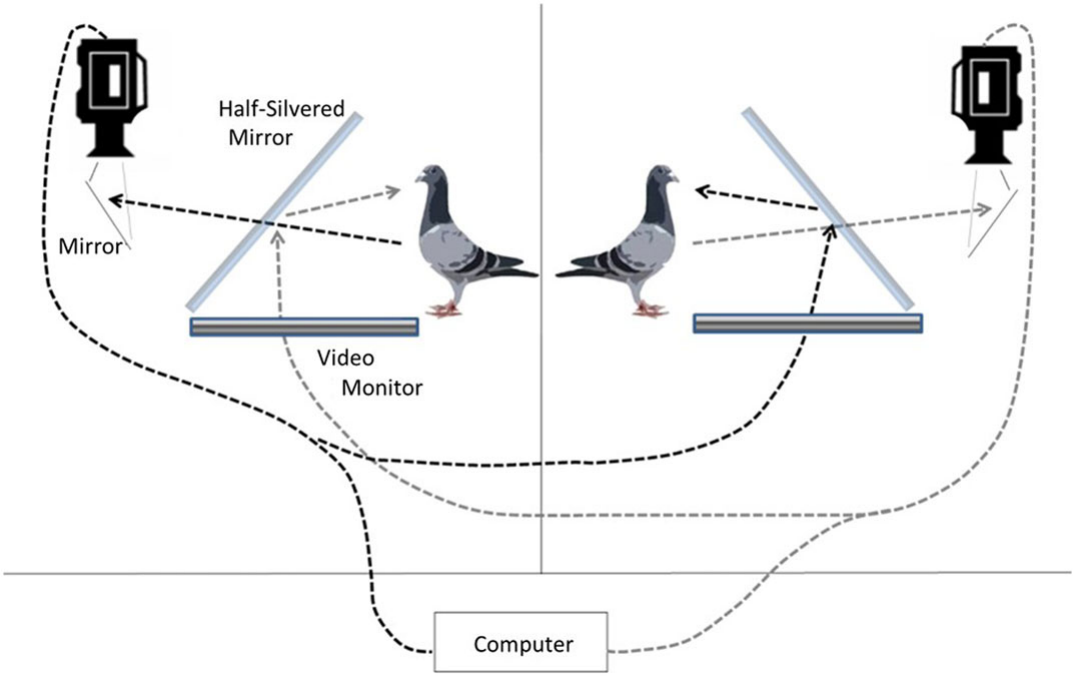
**The double closed-loop teleprompter apparatus.** Two teleprompters enabled live social interaction over a video interface, allowing each subject to be filmed from a hidden camera placed behind the live video image of the other subject. Black dotted and long-dash grey lines denote the course of visual information flow through the video channel from one bird to the other in either direction. The video camera inside the teleprompter films one pigeon off a mirror and through a pane of one-way glass. The video then streams into the control room where the experimenter can observe, and then into the teleprompter apparatus of the other subject.

**Fig. 2.**
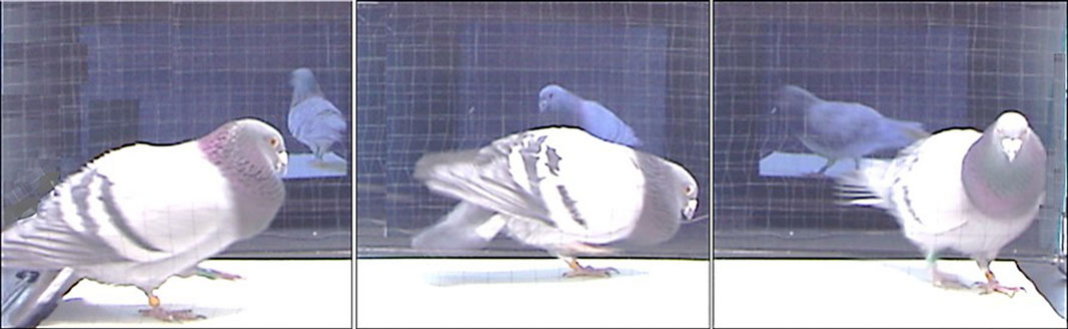
**A male pigeon courting a female partner displayed live in the teleprompter apparatus.** The double teleprompter interface allowed us to film the stimulus partner while they were interacting with a live opposite sex conspecific. When these stimuli were played back in the teleprompter during the experiment, they contained socially motivated behaviour that was oriented directly towards the subject bird at the same position as the original partner.

In the present investigation we test the influence of a video's IPR on the courtship response of male and female pigeons towards opposite sex conspecifics presented on video. We filmed the videos to be used as experimental stimuli in a double closed loop teleprompter apparatus, a setup that enables real time face to face interactive communication (Figs 1,2). During the experiment we will play these video stimuli back to our subjects using only half the teleprompter apparatus. Using a teleprompter setup for filming and playback ensures that the stimuli contain motivated courtship behaviour oriented directly towards the subject animal.

Courtship displays in pigeons involve circle walking, a stylized figure eight walking pattern that contains a rich repertoire of different signals such as charging motions, head nods tail spreads and the ritualized bow-coo display (Goodwin, 1983). The signal content and intensity of circle walking varies in a context dependent and interactive manner, much like a visual conversation (personal observation). The circle walking display typically occurs in short bouts, which vary in duration depending on how many turns are performed. Between each bout there is a resting period where the bird appears to be observing their partner's display and often will signal (e.g. head nod or bow) in response to their partner's display. Female's bouts tend to be relatively short with more resting time, whereas male bouts are more often continuous, sometimes lasting for a full minute or more. In order to allow us to process large amount of behavioural data, we developed an automatic technique for measuring the pigeon's circle walking behaviour using motion energy analysis. We used this automatic coding method to evaluate subjects’ total circle walking duration (TCWD).

By presenting each subject with three different courting opposite sex social partners, and presenting each of these stimuli at either 15 *p*, 30 *p* or 60 *p*, we evaluate 1) how IPR affects TCWD, and 2) whether any effect of social partner on TCWD may be influenced (and confounded) by the effect of IPR.

## RESULTS

Shapiro-Wilk tests for normality do not detect deviations from normality in our data, with the exception of one condition (out of 18). We therefore applied ANOVA to the untransformed data.

An initial 3-way ANOVA on TCWD revealed a main effect of IPR, *F*_2,20_=14.22, *p*<0.001 (partial η2=0.587), a main effect of social partner, *F*_2,20_=8.34, *p*=0.002 (partial η2=0.455), a significant interaction between social partner and sex, *F*_2,20_=6.57, *p*=0.006 (partial η2=0.397), but no main effect of sex.

**Fig. 3.**
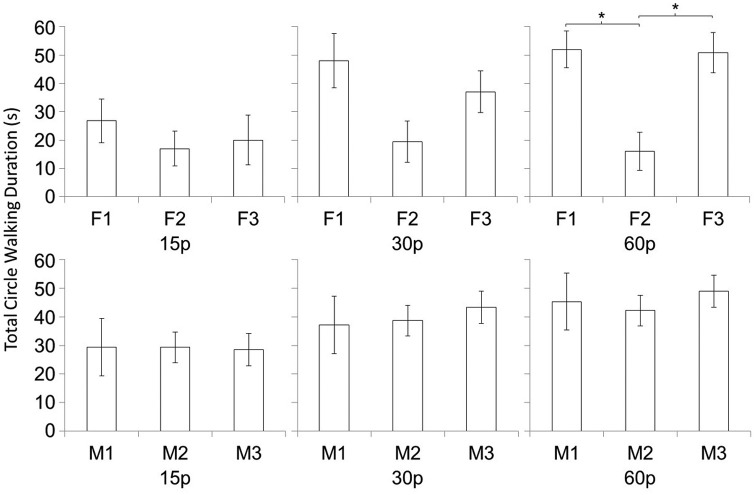
**The interaction between social partner and sex is presented across the different IPR conditions.** As IPR increases, total circle walking duration increases for both males and females. For male subjects (top), the analysis revealed an interaction between the effects of IPR and social partner on male total circle walking duration. For female subjects (bottom) there is no effect of social partner. The asterisk (*) indicates a significant difference (*p*<0.0167) between the marked conditions. Data are means±s.e.m. F1=Female Stimulus 1, F2=Female Stimulus 2, F3=Female stimulus 3, M1=Male Stimulus 1, M2=Male Stimulus 2, M3=Male stimulus 3.

Follow up 2-way ANOVAs conducted for each sex to further explore the interaction between social partner and sex (and its potential impact on IPR) revealed that, in females, there was a main effect of IPR, *F*_2,10_=5.33, *p*=0.027 (partial η2=0.516), but no effect of social partner on TCWD. In males, there was a main effect of IPR, *F*_2,10_=9.71, *p*=0.005 (partial η2=0.660), a main effect of social partner, *F*_2,10_=13.25, *p*=0.002 (partial η2=0.726), and an interaction between the effects of IPR and social partner, *F*_4,20_=2.91, *p*=0.048 (partial η2=0.368) (Fig. 3).

Further analyses conducted to investigate the interaction between social partner and IPR in males revealed that, male TCWD depends significantly on the social partner viewed, but only in the 60 *p* condition, *F*_2,10_=25.14, *p*<0.001, partial η2=0.834. Within the 60 *p* condition *t* tests revealed that males responded less towards F2 than F1 and F3 (*t*_5_=5.17, *p*=0.004, *t*_5_=4.88, *p*=0.005, respectively). Error probabilities remain statistically significant even under Bonferroni correction for three possible comparisons (adjusted α=0.017). Cohen's effect size values comparing males responses between F2 against F1 and F3 show large effect sizes for both comparisons (*d*=2.21 and *d*=2.05 respectively). It was clear from visually inspecting the male data that all 6 males were not responding to the female stimulus F2. This was creating a floor effect and preventing a clear picture of IPR effects.

**Fig. 4.**
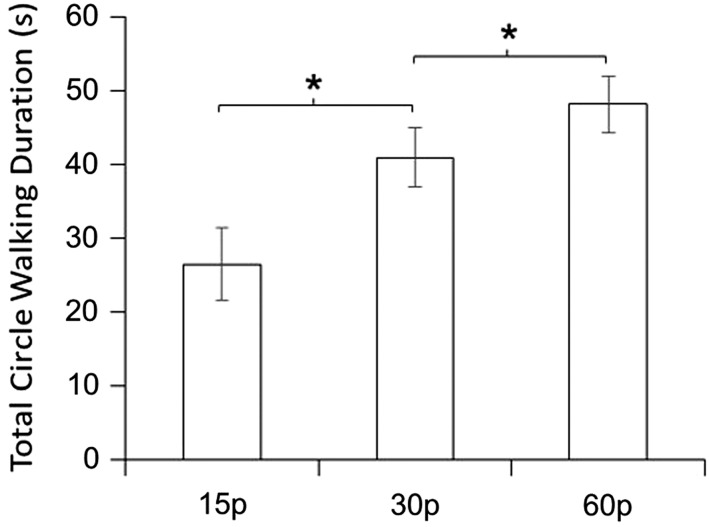
**Total circle walking duration (TCWD) is significantly affected by image presentation rate (IPR).** The response differences towards videos presented at 15 *p*, 30 *p* and 60 *p* implies that each stimulus contains a different set of motion cues, resulting in three different levels of behavioural response. Data are means±s.e.m. The asterisk (*) indicates a significant difference (*p*<0.0167) between the marked conditions.

To inspect the main effect of IPR further, we removed all trials using the F2 stimulus and ran the analysis (3-way ANOVA 3[IPR]×3[social partner]×2[sex]) a second time on male and female data combined. The main effect of social partner disappeared. The main effect of IPR on TCWD became much more apparent, *F*_2,20_=17.63 *p*=0.001, partial η2=0.635 (Fig. 4). Further Bonferroni corrected *t* tests (α=0.017) revealed that TCWD increased across all three conditions, from the 15 *p* to the 60 *p* condition, *t*_11_=−4.31, *p*=0.001, from the 15 *p* to the 30 *p* condition, *t*_11_=−4.52, *p*=0.001, and from the 30 *p* to the 60 *p* condition, *t*_11_=−2.91, *p*=0.014. Cohen's effect size values for the 15 *p,* 30 *p* comparison (*d*=0.928) and the 30 *p*, 60 *p* comparison (*d*=0.5273) revealed a large and moderate effect size respectively. The main difference from the first analysis is that, once F2 was removed, the differences between 15 *p*, 30 *p* and 60 *p* all became more distinctive.

## DISCUSSION

When we showed pigeons moving images of courting opposite sex conspecifics, IPR – the rate at which images were displayed on screen – had a significant effect on the pigeon's behavioural response towards those images. Furthermore, the response rates were distinct across IPR conditions (15 *p*, 30 *p* and 60 *p*), suggesting that in each condition there is a different set of behaviourally relevant cues falling above and below the pigeon's effective CSF. In males we also found an effect of social partner on circle walking behaviour as well as an interaction between social partner and IPR. Males consistently responded much less to one of the female partners (F2) compared to the other two (F1 and F3). However, the effect of social partner on behaviour only became significant in trials using the 60 *p* stimuli. This interaction serves to demonstrate how the effects of insufficient IPRs on behaviour can obscure and confound other experimental effects of interest such as individual differences between social partners. This interaction effect between a video artefact and a behaviourally relevant variant corroborates previous predictions by several authors (e.g. Fleishman and Endler, 2000; Swaddle et al., 2006).

The smoothness and completeness of conspecific motion patterns may be important for several reasons. Here we explore some possible candidates for motion-based cues that could explain the present data. These include cues for biological motion perception, cues that guide attention and promote engaged interaction, and cues that function in social evaluation and preference behaviour.

The behaviour of social animals is thought to be innately wired to quickly orient towards biological motion (Johnson, 2006; Regolin et al., 2000; Troje and Westhoff, 2006; Vallortigara et al., 2005). Recent work suggests that the brain has early visual filters attuned to the local acceleration profiles characteristic of an animate self-propelled agent (Chang and Troje, 2008). These ‘life’ detectors can function to elicit what has been called the ‘visual grasp reflex’, which rapidly brings behaviourally relevant movements into the central focus for visual processing (Fleishman, 1988). The rise and fall of the foot under gravitational constraints, for instance, exhibits a signature dynamic that the brain can exploit to quickly determine the presence and walking direction of another animal (Saunders et al., 2009). The pigeon's typical walking frequency is approximately between 3–8 Hz (Troje and Frost, 2000) and therefore even the most basic signatures of the gait cycle likely appeared undersampled in the 15 *p* condition. Research investigating these specialized early ‘life’ detectors in humans suggests that information is retrievable in local motion segments as short as 100 ms (Chang and Troje, 2009). Signatures based on acceleration patterns require at least three frames within that window and therefore a minimal IPR of 30 Hz. Given the faster visual system of birds, it is possible that they are sensitive to such information on an even smaller time scale but to demonstrate that sensitivity in response to video, IPRs have to be high enough. If such perceptual triggers are not available a slowed, stunted or absent behavioural response would result.

Motion cues play an integral role in guiding visual attention during social interactions. Fleishman (Fleishman, 1988) suggests that many animal signals are explicitly designed to exploit the signal receiver's ‘visual grasp reflex’. Song birds, for example, frequently exhibit rapid visual gestures such as wing flicks or head movements during vocal interactions (Todt and Fiebelkorn, 1980; West and King, 1988; Williams, 2001). These interactive visual signals are hypothesized to increase the efficiency of vocal transmissions by directing visual attention toward the signaller and by signalling responsiveness and engagement during the dialogue. Because they are designed to draw attention such ‘look this way’ gestures are typically characterized by abrupt and rapid temporal profiles (waving, snapping and flicking) so that they can be easily picked out from a noisy visual background. In many cases, such signals are not only quick but also characteristically subtle, as they are designed for private conversation and to be detected only by an attentive social partner, akin to a visual whisper. West and King (West and King, 1988), for instance, measured the wing flicks of cowbird mothers (which are thought to act as critical feedback in juvenile song development) to be approximately 200 ms in duration. Because of their characteristically rapid and often subtle features, these cues are particularly vulnerable to motion undersampling and distortion. If we apply the Nyquist Theorem to the cowbird's wing flick for instance, it would take a sampling rate of 10 Hz to sample even the most basic periodicity of this movement. Any smaller-scale motion components within the wing stroke would be vulnerable to temporal undersampling. Nuances would be lost. We can speculate that a selective breakdown in the quality of attentional triggers in the images displayed at low IPRs caused a deterioration in visual focus and engagement towards the partner's display and, in turn, a deterioration of the behavioural response.

In addition to guiding attention, research has suggested that motion is important for social evaluation and preference behaviour. Mounting evidence suggests that, in many avian species especially, observers rely on very fine variations in the speed, agility and forcefulness of courtship actions to make mate choices for reproduction (Barske et al., 2011; Byers et al., 2010; Fusani et al., 2007). For example, Fusani et al. (Fusani et al., 2007) used high speed video (125 frames per second) to inspect male Golden-collared manakin (*Manacus vitellinus*) courtship displays. They were able to document and study a number of rapid visual signals that they could not capture with traditional camera equipment. Among the motion cues observed, they found significant individual variation in the duration of males’ courtship jumps on the order of 10-50 ms. To put this in perspective a periodic movement on this time scale would, according to Nyquist, require a sampling frequency between 40 and 200 Hz to fully capture the individual variations in movement. The existence of individual phenotypic variation of a sexual trait is generally thought to indicate its relevance as a behaviourally relevant signal that is likely to be used in assessing mate quality. The fact that high speed cameras (125 frames per second) were required to measure these small scale individual differences in movement implies that our traditional video technology is not sufficient to capture this type of variation. The variation of courtship response across IPR conditions in the present study might be explained by a decreased ability to assess the fine-scale variation indicative of display quality and driving social preference behaviour. The result that males discriminated between individual social partners presented at 60 *p* but not at 15 *p* or 30 *p* provides some evidence that, for courting pigeons, the cues advertising quality and promoting preferential responding lie somewhere around the order of 30 Hz (thereby requiring 60 *p* IPR to represent the motion).

It is important to acknowledge that the temporal resolution of the pigeon's visual system may exceed the 60 Hz value. The absence of IPR conditions above this value represents a limitation to this study. The bandwidth capacities of the video technology used to film the stimuli and display the stimuli in a progressive format limited our scope in this regard.

What are the risks of studying social behaviour with stimuli that inaccurately represent motion? Our data demonstrate the potential for confound arising from an interaction between the effects of IPR and the effects of the social partner on behaviour. In this case, the effect of social partner on male behaviour was not captured at 15 *p* and 30 *p* IPRs. This appears to be a false negative finding, given that significant differences were apparent in the 60 *p* condition.

Another possibility is that distortions arising from low IPR could give rise to spurious positive results. Any video playback study would be at risk of this if motion quality either characterizes or co-varies with the variable that is being experimentally manipulated. A previous study found that female sticklebacks decreased their response behaviour towards male courtship displays presented at triple tempo as compared to stimuli presented at normal or double tempo (Rowland, 1995). Rowland interpreted these findings to represent a female preference for normal and double tempo male displays over triple tempo displays. That might well be the case, but in light of our discussion here, Rowland's results could also be an artefact. An alternative explanation is that the male's motion was undersampled and appeared distorted in triple tempo conditions but was sufficiently sampled and appeared natural at lower speeds.

Other potential confounds may not be so obvious. CSF depends on the motion dynamics of the partner being viewed. Motion dynamics, in turn, depend on several social variables such as species, action type, sex, age or dominance. Furthermore, the motion cues that the subject animal attends to will likely depend on behavioural and social context, such as the age, sex, familiarity and dominance of the partner. It follows that the subject's effective CSF may vary across such social variables, which could result in complex confounds and errors. For example, a handful of studies have found that subjects’ social preferences, their tendency to respond more to certain types of partners over others, sometimes appear to reverse or change in strength when the social choices are presented on video, as compared to when they are presented in live form. For example, female zebra finches (*Taeniopygia guttata*) prefer their mates over other males when greeting these males live, but behave indiscriminately towards their mates and other males if they are presented on video (Swaddle et al., 2006). Male *Anolis* lizards (*A. cybotes and A. marconoi*) are equally aggressive towards males of their own species and males of other species, but when these opponents are displayed on video male lizards aggress more towards the members of their own species (Macedonia et al., 1994). Female swordtail fish (*Xiphophorus helleri*) prefer males with long tails to a greater extent if these males are shown on video than if the males are presented live (Trainor and Basolo, 2000). While there are several possible explanations for such unexpected results, including the confounding effects of other video artifacts such as colour, depth or social interactivity (Zeil, 2000), one is that the impact of impoverished motion cues on social preference behaviour varies non-uniformly across partner types.

While many of the researchers in the previous examples have speculated that the unexpected effects in their studies may arise from video artifacts, our study represents one of the few demonstrations of such an occurrence. The lessons learned here are not limited to motion artifacts. This study can serve as a demonstration of the impact confounding effects might have when dealing with other video artifacts such as colour, depth, and social interactivity. Demonstrating how confounds work to skew experiments will not only enable more carefully controlled experiments in the future, but can also teach us about the natural functions of the complicating factors in real-world social interaction. For instance, in this case, we show that motion likely plays a role in discriminating between conspecifics, and we suggest some potential perceptual and behavioural functions through which this effect occurs.

Is video playback still a useful tool for researchers? We believe the answer is yes. While results like these may dissuade others from employing these methods, this technology still represents an improvement over robotic or computer-animated stimuli in recreating, manipulating and controlling naturally moving stimuli for studying animal behaviour. These alternate methods rely on humans to approximate the natural form and behaviour of the animal, which undoubtedly results in a greater number of potential confounding artifacts than video. Progress in the field of video playback has the potential to become an unprecedented source of discovery into animal behaviour and neuroscience.

We have several recommendations for researchers to avoid confounds, although each study species and each study variable of interest carries its own unique set of considerations. Fortunately, video technology is advancing at a fast pace. Consumer video cameras and industrial cameras are now available with much higher frame rates. Likewise, computer screen are capable of displaying at 120 fps and above. Researchers should use the highest frame rate available on their recording equipment. Researchers should also be sure to report the frame rate in the methods section of their study. Furthermore researchers should consider behavioural motion patterns that might co-vary with their experimental variable either in form or in the degree of behavioural relevance the motion pattern has to the subject. For instance, variables such as attractiveness, social partner, intra-specific vs inter-specific, sex, age, dominance and familiarity all represent variables that may impact either the quality of the motion patterns observed or the degree to which the subject will attend and react to those particular motion patterns during the experiment, or both. If the variable of interest is something where motion cues or the behavioural relevance of motion cues may vary, consider what direction you might expect any spurious effects to be in; then, take this into account when interpreting your results. Researchers should consider standardizing stimuli according to the amount, type and quality of motion that occurs in the video. We present in our methods a technique for automatically coding behaviour using EyesWeb Analysis. This tool can also be used to ensure that there is equal motion energy across all stimuli.

Video playback research takes us one step closer towards gaining an in-depth understanding of the meaning of dynamic visual signals and the mechanisms by which they are perceived. However, it is crucial that we understand whether non-human subjects actually experience a video partner as a naturally moving social partner, containing all the attention-grabbing and socially engaging cues characteristic of real live social stimuli. If we do not know to what degree video generates a realistic motion percept, we may be presenting stimuli that are distorted in the very feature that video playback so valuably enables us to control and study.

## MATERIALS AND METHODS

### Subjects

A total of six male and six female homing pigeons (*Columba livia*) were selected from a pigeon aviary, 23 m², containing a colony of 70 pigeons assembled from racing breeders in the Kingston area of Ontario, Canada. Birds were selected to participate in the study if they exhibited active courtship and maintained it under experimental conditions. Beginning two weeks prior to the study, the subjects were housed individually in standard steel rabbit cages (60×46×40 cm) so that they were visually isolated from the other birds in the room. Birds were kept on a feed of cracked corn and standard pigeon grains. Their light cycle was kept such that it approximated Eastern Ontario natural dawn-dusk light cycle. Our animal care protocol was reviewed by the Queen's University Animal Care Committee to ensure adherence to all relevant institutional and national animal welfare laws, guidelines and policies.

### Stimuli and Apparatus

Stimuli were moving images of three males (M1, M2 and M3) and three females (F1, F2, F3) that were filmed during a live courtship interaction. The videos were manipulated such that they played back at different IPRs: 15 *p*, 30 *p* or 60 *p*. Each of the 12 subjects viewed nine different video playback stimuli, three social partners displayed at each of the three IPR conditions. This design yielded a total of 108 experimental trials.

All stimuli depicted an opposite sex conspecific engaged in the first minute of a live courtship interaction, mediated by a double closed loop teleprompter interface. This setup enables live interaction from two remote locations (Figs 1,2). Footage was captured at 60 *p* using the Dragonfly2 video camera and FlyCapture software (Point Grey Research Inc., Richmond, BC, Canada).

The double teleprompter consisted of two teleprompter setups in two different rooms. Each consisted of a 19″ Samsung LCD Syncmaster 1701 monitor which laid flat and faced up towards a half-silvered mirror (64×55 cm) mounted at a 45° angle with respect to the horizontal plane. The Dragonfly 2 camera was placed directly behind the half-silvered mirror and fixed so that it pointed vertically downwards inside the teleprompter, filming the subject bird at eye level by way of a small mirror placed a few inches below the camera (45° to the camera and 45° to the bird). The purpose of this mirror was to compensate for the mirror flip that occurs on the half-silvered glass when the video image is projected. The teleprompter was housed in an aluminium frame (60 cm wide×64 cm tall×67 cm long). To make the interior of the teleprompter dark, black Choroplast plastic board was used to cover the top and the sides of the apparatus. This ensured that the subject bird saw only the reflection of the other bird on the half-silvered mirror and could not see the interior of the teleprompter where the video camera was housed. The same black Choroplast board was used as a background placed behind each stimulus bird as it was being filmed. While in the teleprompter setup, the subjects were placed in 46×46×46 cm cages made of a thin steel framing and covered with walls made of mist netting. The camera and teleprompter setup were calibrated to make the playback image appear life-size. The final image was projected approximately 80 cm away from each pigeon, as measured at eye level to the approximate location where the image would appear after being reflected onto the half-silvered mirror.

We took great care to deliver the stimuli such that they were guaranteed to display at the specified frame rates. We first rendered 60 frames per second of uncompressed images. We then used Matlab und the Psychophysics Toolbox (Brainard, 1997) to synchronize the presentation of the single images with the 60 Hz refresh rate of an LCD computer screen. For the 60 Hz IPR condition we switched images at every screen refresh. For the 30 Hz IPR condition, we switched images only every other screen refresh (and using only every second frame of the recorded image sequence). Likewise, for the 15 Hz IPR condition, we switched images only every four screen refreshes.

During the experiment only half of the double teleprompter setup was used to display stimuli, and thus the courtship response elicited from subjects was not interactive (as it had been during stimulus creation). Each trial lasted one minute. The final one-minute stimuli that were selected for the experiment all contained similar amounts of courtship behaviour, between 40 and 45 s of circle walking display. At no stage did we ever compress the videos in any way. For each base stimulus, the image content and quality was identical across all three IPRs; the only difference was the rate at which the image was updated on the screen.

### Procedure

Before the experiments began, birds were habituated for 30 minutes daily to the experimental apparatus until they appeared comfortable and responded with courtship behaviour to videos of conspecifics. During a typical experimental trial the subject was placed in the teleprompter apparatus and the monitor was switched on at trial onset (see supplementary material Movie S1). No bird was run more than once every 4 hours and the experiments always took place between 8 am and 6 pm. Ambient noise (a radio receiver running at moderate volume) was used to mask environmental noise during the experiment. An observation camera was placed in the room to record the subject's behaviour for coding and analysis.

### Automatic Behavioural Coding

We developed an automatic method of coding behaviour using EyesWeb Open Software Platform Motion Analysis Library (Camurri et al., 2004; www.eyesweb.org), which provides a set of tools for analysing motion energy in video footage using optical flow analysis algorithms. When the pigeon circle walks the motion energy values obtained from video are elevated with respect to the motion energy values obtained when the pigeon is not circle walking. In this way, we could convert measures of motion energy into a measure of the courtship response.

The EyesWeb procedure first converted the video to grey scale, and then computed a value for the vertical and horizontal optical flow at each position in the image over each frame transition, using the Lucas–Kanade method (Lucas and Kanade, 1981). The matrix of optical flow values were squared and averaged, producing a single motion energy value for each frame transition in the video.

The time series of motion energy values for each video was then smoothed and thresholded. We used a smoothing kernel of 23 frames (averaging over a window spanning the 11 preceding frames and the 11 following frames for each individual value) and an empirically set threshold which discriminated clearly between inactive and active periods. The time-series of the binary motion energy values was then processed to evaluate our behavioural measure: total circle walking duration.

We tested our automatic coding technique by using a separate data set that had been manually coded using the Queen's Video Coder Software (Baron et al., 2001) and Sony Sound Forge for the audio component. The data set contained 360 audio/video files containing footage of 6 different pigeons responding to opposite sex conspecifics in the teleprompter apparatus. The data was collected as part of a research study with 5 experimental conditions (unpublished). The pigeon behaviours coded by duration included circle walking, standing, walking and the behaviours coded by frequency were coos, bows, tail drags and preens. Circle walking was coded only if the bird completed a full 360° circle or figure eight. If the bird stopped for at least 3 s and then restarted circle walking, a new circle walking bout was coded. The four raters, the experimenter and 3 trained undergraduate volunteers, were always blind to experimental condition. Each video was rated by at least two raters yielding an average Pearson's correlation coefficient of *r*=0.86 (*p*<0.01) for the total scores of all behaviours tested (supplementary material Table S1).

There was a strong positive correlation between TCWD (s) measured automatically (motion energy above threshold) and TCWD (s) measured manually, *r*_358_=0.936, *p*<0.01. A scatterplot summarizes this result (supplementary material Fig. S1). A table demonstrating correlation coefficients between the automatic measure and various manually coded courtship behaviours is provided (supplementary material Table S1).

## Supplementary Material

Supplementary Material
