# Supplementary material for: The influence of motion quality on responses towards video playback stimuli
Source: Biol Open. 2015 May 11;4(7):803–11. doi: 10.1242/bio.011270 (PMC4571084; doi:10.1242/bio.011270)
Supplement: Supplementary Material [file supp_4_7_803__index.html]

The influence of motion quality on responses towards video playback stimuli — The influence of motion quality on responses towards video playback stimuli — Supplementary Material 

# The influence of motion quality on responses towards video playback stimuli

## BIO011270 Supplementary Material

- Supplementary Material
