# Supplementary material for: The influence of motion quality on responses towards video playback stimuli
Source: Biol Open. 2015 May 11;4(7):803–11. doi: 10.1242/bio.011270 (PMC4571084; doi:10.1242/bio.011270)
Supplement: Supplementary Material [file supp_bio.011270_BIO011270supp.pdf]

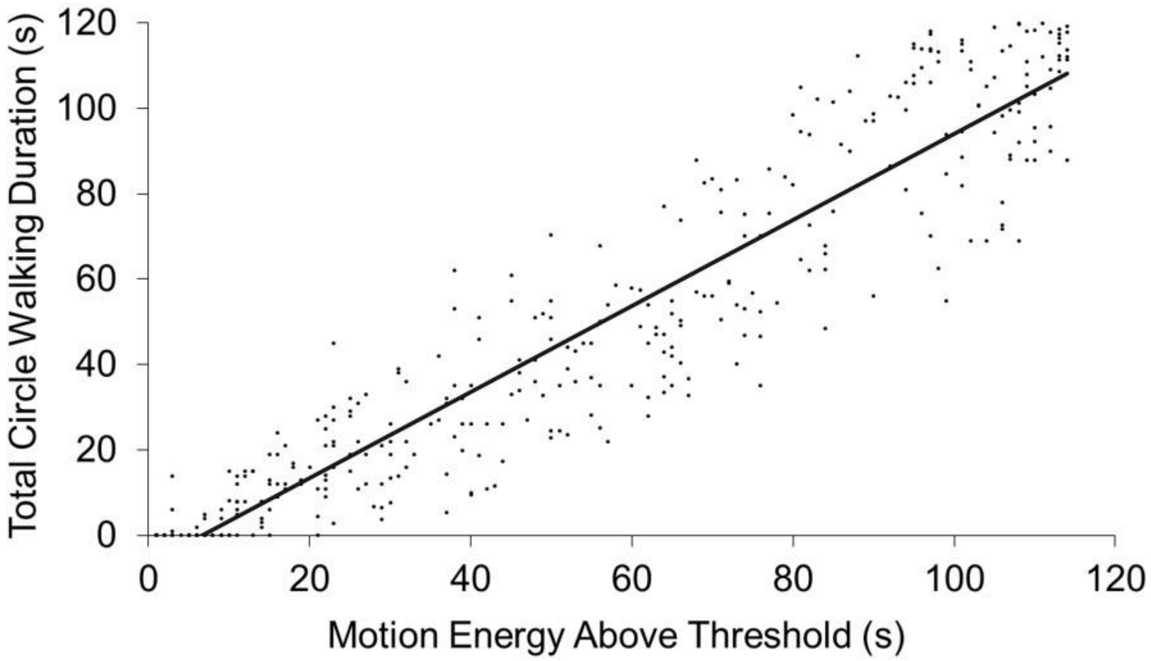

Supplementary material Fig. S1. The correlation between motion energy above threshold and circle walking duration. There is a strong positive correlation (Pearson's correlation coefficient, r = 0.936) between the automatically coded measure of motion energy above threshold (s) and the manually coded scores of circle walking duration (s). Motion energy above threshold represents a measure of circle walking duration. This measure is obtained by quantifying the motion energy of behaviour captured on video.

Supplementary material Table S1. The relationships between measures of courtship behaviour. Pearson correlation coefficients between the measures of motion energy above threshold (s), the duration of circle walking (s), number of bows, number of coos, number of tail drags, the duration of standing (s), the duration of walking (s) and the number of preens are listed in the table. Motion energy above threshold represents an automatic measure of circle walking duration using motion energy analysis. All other behavioural measures were obtained with manual coding methods. The double asterisk (**) indicates significance at the 0.01 level, n = 360.

	Motion Energy	Circle Walk	Bow	Coo	Tail Drag	Stand	Walk	Preen
Motion Energy	1							
Circle Walk	.936**	1						
Bow	.724**	.708**	1					
Coo	.603**	.609**	.801**	1				
Tail Drag	.589**	.560**	.498**	.490**	1			
Stand	-.924**	-.956**	-.739**	-.616**	-.571**	1		
Walk	-.008	.003	-.071	-.083	-.061	-.009	1	
Preen	-.480**	-.458	-.444**	-.329**	-.268**	.552**	-.103	1

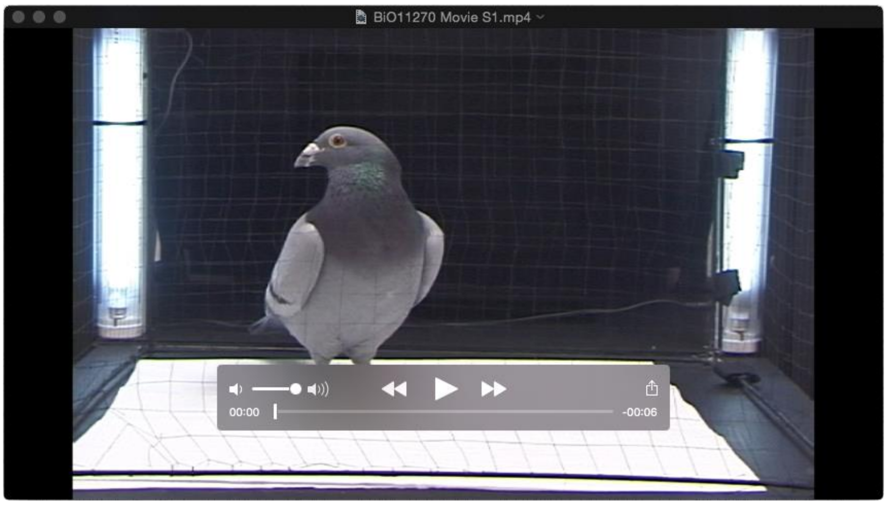

Supplementary material Movie 1. An example of a male pigeon responding with circle walking behaviour in the teleprompter apparatus towards a video playback of a female pigeon performing a courtship display.
